# Seeking neutral: A VR-based person-identity-matching task for attentional bias modification – A randomised controlled experiment

**DOI:** 10.1016/j.invent.2020.100334

**Published:** 2020-08-14

**Authors:** Lichen Ma, Anne-Wil Kruijt, Anna-Karin Ek, Gustaf Åbyhammar, Tomas Furmark, Gerhard Andersson, Per Carlbring

**Affiliations:** a Department of Psychology, Stockholm University, Frescativägen, 114 19 Stockholm, Sweden; b Department of Psychology, Uppsala University, P.O. Box 256, 751 05 Uppsala, Sweden; c Department of Clinical Neuroscience, Karolinska Institute, Solnavägen 1, 171 77 Solna, Stockholm, Sweden; d Department of Behavioral Sciences and Learning, Linköping University, 581 83 Linköping, Sweden

**Keywords:** Attentional bias, Attentional bias modification, Social anxiety, Virtual reality, Dot-probe, Person-identity-matching

## Abstract

**Background:**

Attentional bias modification (ABM) aims to reduce anxiety by attenuating bias towards threatening information. The current study incorporated virtual reality (VR) technology and 3-dimensional stimuli with a person-identity-matching (PIM) task to evaluate the effects of a VR-based ABM training on attentional bias and anxiety symptoms.

**Methods:**

One hundred participants with elevated social anxiety were randomised to four training groups. Attentional bias was assessed at pre- and post-training, and anxiety symptoms were assessed at pre-training, post-training, 1-week follow-up, and 3-month follow-up.

**Results:**

Change in anxiety did not correlate with change in bias (*r* = −0.08). A repeated-measures ANOVA showed no significant difference in bias from pre- to post-ABM, or between groups. For anxiety symptoms, a linear mixed-effects model analysis revealed a significant effect of time. Participants showed reduction in anxiety score at each successive assessment (*p* < .001, Nagelkerke's pseudo *r*^2^ = 0.65). However, no other significant main effect or interactions were found. A clinically significant change analysis revealed that 4% of participants were classified as ‘recovered’ at 3-month follow-up.

**Conclusions:**

A single session of VR-based PIM task did not change attentional bias. The significant reduction in anxiety was not specific to active training, and the majority of participants remained clinically unchanged.

## Highlights

• First attentional bias modification training (person-identity-matching) with 3D stimuli
• No bias was observed at baseline, and no change detected post-training.
• No correlation between attentional bias and anxiety observed.
• Reduction in anxiety observed regardless of training contingency or stimuli used.
• Only a small percentage of anxiety reduction achieved was clinically significant.

## 1 Introduction

Social anxiety disorder (SAD) is a common mental health problem that impairs social functioning and reduces quality of life (Barrera and Norton, 2009; Saris et al., 2017). Both psychological treatments such as cognitive behavioral therapy (Carpenter et al., 2018) and pharmacological treatments (Jakubovski et al., 2019) for SAD have shown high efficacy and can result in positive long-term outcomes (Mayo-Wilson et al., 2014). However, barriers such as inaccessibility to therapy (e.g. therapist shortage or geographical distance), high cost, long wait time, and stigmatisation can prevent SAD sufferers from seeking treatment (Hedman et al., 2016). In order to overcome these barriers, there has been an ongoing effort to develop treatment options that are accessible, effective, and acceptable for SAD patients (Heeren et al., 2015b; Lindner et al., 2017).

### 1.1 Attentional bias modification

Attentional bias modification (ABM) for SAD operates on the assumption that dysfunctional anxiety is caused by the preferential allocation of attention towards socially threatening information (Cisler and Koster, 2010; McNally, 2018). A number of studies have reported such an attentional bias in anxious individuals (e.g. Amir et al., 2008; Andersson et al., 2006; De Voogd et al., 2014). The rationale for ABM is that if the attentional bias underlying problematic anxiety can be attenuated via training, there will also be an associated reduction in anxiety symptoms (Bar-Haim, 2010; Koster et al., 2009; MacLeod and Mathews, 2012).

The effectiveness of ABM as a viable treatment option for anxiety disorders remains a contentious topic despite the large body of literature (see Cristea et al., 2015, Cristea et al., 2017; Grafton et al., 2017; Kruijt and Carlbring, 2018; McNally, 2018; Mogg and Bradley, 2016). While early studies in the field have reported significant reduction in attentional bias and anxiety symptoms following ABM training (e.g. Amir et al., 2008; Dandeneau et al., 2007; MacLeod et al., 2002), more recent studies have failed to replicate these results (e.g. Boettcher et al., 2013; Carlbring et al., 2012; Heeren et al., 2015a; Ma et al., 2019). Meta-analyses on ABM studies have produced conflicting findings, with some researchers concluding ABM to be an effective therapeutic tool for anxiety disorders (Linetzky et al., 2015), while others question the reliability and validity of the existing evidence supporting such a claim (Cristea et al., 2015).

### 1.2 Innovative ABM

Proponents of ABM maintain that anxiety symptom change cannot occur without a change in attentional bias. Therefore, the task used (the *procedure* of ABM) must successfully modify attentional bias (the *process* of ABM) in order for the training to be of any therapeutic value (Grafton and Macleod, 2016; Grafton et al., 2017). The most commonly used task in ABM research is the dot-probe task (MacLeod et al., 1986), which serves both as a measurement task and a bias modification task (by introducing a training contingency that encourages a shift in attention away from threatening information). However, in light of inconsistent results from studies using the dot-probe task as the ABM training procedure, many researchers have emphasised the need to develop and validate alternative ABM tasks (Bar-Haim, 2010; Van Bockstaele et al., 2014). One criticism against the dot-probe task is that it is very repetitive. The concern is that if the participant loses focus during the ABM training, they will be less likely to achieve bias modification and symptom reduction (Heeren et al., 2015b).

One way to improve ABM is to make the training more dynamic and engaging. Notebaert et al. (2015) developed the person-identity-matching (PIM) task based on the card game ‘Snap’, where participants were asked to make a judgement on whether two faces displaying the same expression (angry or happy) belong to the same individual. In the attend-happy task, participants were instructed to only focus on the identities of the happy faces to encourage attentional shift away from threat. In the attend-angry task, participants were asked to focus their attention on the angry faces instead. The PIM task also differed from traditional dot-probe tasks in that feedback of the correct response was provided to the participant. The authors reported that after ABM training using the PIM task, participants in the attend-happy condition showed reduced attentional bias towards threat compared to those in the attend-angry condition. Furthermore, participants in the attend-happy condition also showed less negative mood shift in a stressor task compared to those in the attend-angry condition.

Another potential strategy to increase task engagement is the incorporation of new technology. Urech et al. (2015) carried out a proof of concept study where ABM training was delivered inside a virtual environment. This virtual reality (VR) based ABM successfully induced a shift in attentional bias, along with a reduction in anxiety. VR-based treatment provides a great deal of control, since the experimenter can modify the therapy environment and how the stimuli are presented at will. The highly controlled environment also ensures consistent delivery of the treatment. The immersive nature of the VR environment can potentially increase task engagement and ecological validity. Furthermore, if a VR-based treatment can achieve equal clinical outcomes as face-to-face therapy, the non-reliance on clinician coupled with increasing accessibility of VR programs could mean wider distribution and lower costs compared to treatment at a clinic (Lindner et al., 2017).

### 1.3 Current study

The aim of the current study was to test the effectiveness of a single-session, VR-based PIM task in reducing attentional bias and social anxiety in participants with elevated trait anxiety recruited from the general population. Participants were randomly assigned to one of four experimental groups undergoing PIM training towards either neutral or disgust, with either 2D or 3D stimuli. Attentional bias was measured pre- and post-training using a dot-probe task. Self-reported anxiety symptoms were assessed at pre-training, immediately post-training, at 1-week follow-up, and at 3-month follow-up. We hypothesised that, at post-training and follow-up assessments, (i) participants in the neutral PIM groups would have lower attentional bias and anxiety scores compared to those in the disgust PIM groups; (ii) participants who received training with 3D stimuli would have lower attentional bias and anxiety scores compared to those who received training with 2D stimuli.

## 2 Method

### 2.1 Participants

One hundred participants were recruited from the general population between June and October 2017. The study was advertised on websites, newspapers, and national radio. Potential participants were directed to visit the study website iTerapi (Vlaescu et al., 2016), where they could learn more about the study and register an account to be screened for eligibility.

Inclusion criteria were: (i) score 30 or above on the Liebowitz Social Anxiety Scale, self-report (LSAS-SR), indicating probable SAD (Rytwinski et al., 2009); (ii) normal depth perception; (iii) fluent Swedish speaker; and (iv) at least 18 years of age. Exclusion criteria were: (i) any psychological treatment/counselling within the past 90 days; (ii) any change in psychopharmacological medication within the past 90 days (with the exception of as-needed medications such as beta-blockers); and (iii) Depression and suicidal ideation (as indicated by a total score of 14 or higher, and/or a score greater than 0 on the suicide item of the Patient Health Questionnaire (PHQ-9; Kroenke et al., 2001)). The study was approved by the Regional Ethical Review Board in Stockholm, Sweden.

### 2.2 Self-reported measures

The primary outcome measure was social anxiety assessed by the LSAS-SR (Fresco et al., 2001). The LSAS-SR is a 24-item questionnaire that taps into two dimensions of social anxiety: performance anxiety (13 items) and social situations (11 items). Participants first indicated how much fear is associated with the situation described by each item using a 4-point Likert scale. The same 24 items were rated again to indicate how much avoidance is associated with each situation. The LSAS-SR has demonstrated good test-retest reliability, structural validity, and internal consistency (Baker et al., 2002).

Secondary outcome measures included the Patient Health Questionnaire (PHQ-9; Kroenke et al., 2001) for depression, Generalised Anxiety Disorder 7-item scale (GAD-7; Spitzer et al., 2006), the Difficulties in Emotion Regulation Scale-16 (DERS-16; Bjureberg et al., 2016), and Brunnsviken Brief Quality of Life Inventory (BBQ; Lindner et al., 2016). All measures were in Swedish. The DERS-16 and BBQ were originally developed in Swedish. Translated versions of the LSAS-SR, PHQ-9, and GAD-7 have all been validated and used in previous studies on clinical populations (e.g. Hansson et al., 2009; Hedman et al., 2010; Johansson et al., 2013).

### 2.3 Attentional bias assessment and modification program

#### 2.3.1 Apparatus

The VR-ABM program was developed by Mimerse (https://mimerse.com). The VR hardware used was the Oculus Rift consumer version headset, and response input was recorded using a wired Xbox 360 controller. The experiment ran on a Corsair Tortuga computer with 4Ghz Intel Core i7 processor and NVIDIA GeForce GTX 1080 graphics card.

#### 2.3.2 Stimuli

The facial stimuli used in the current study were selected from the BP4D-Spontaneous Database (Zhang et al., 2014). A total of 32 individuals (50% female) each showing a neutral expression and a disgusted expression were included in the stimuli set, with a total of 64 expressions. Two sets of stimuli were created from these 64 images, one set being two dimensional (2D) and another set three dimensional (3D). The 2D images have a resolution of 1040 × 1392 pixels.

Meta-analytical studies have reported mixed findings regarding the moderating effect of stimulus modality on ABM (Jones and Sharpe, 2017). For instance, some studies found pictures to be more effective than words in changing bias (Beard et al., 2012), while others have found the opposite (Hakamata et al., 2010). Different facial expressions have been used in ABM studies, including anger, sadness, and disgust. The decision to use disgust as the socially threatening facial expression was because disgust underpins many complex emotions that are closely associated with social anxiety, such as shame, humiliation, and rejection (Amir et al., 2003; Phillips et al., 1998).

#### 2.3.3 Attentional bias measurement (dot-probe task)

**Fig. 1:**
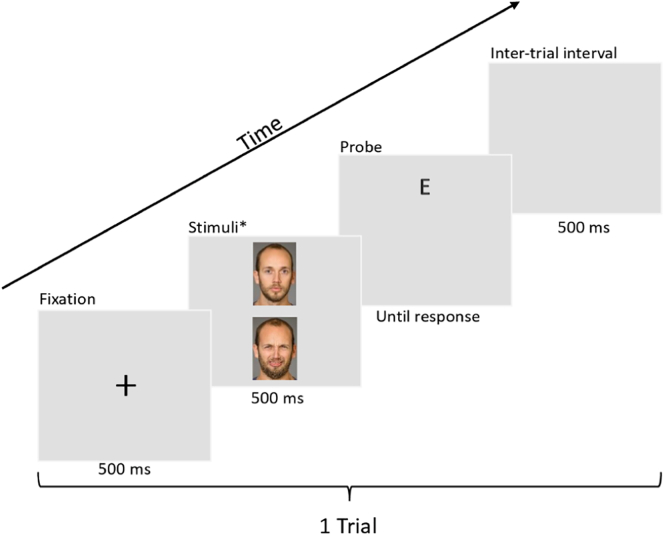
Example trial of a dot-probe task. Each trial began with a fixation cross appearing on screen for 500 ms. After the fixation cross, two faces from the same individual showing a neutral expression and a disgusted expression appeared on the screen (arranged vertically) for 500 ms. The position of the neutral/disgust expressions was counterbalanced, so that each expression appeared with equal frequency on top or bottom. After the faces disappeared, a probe (letter ‘E’ or letter ‘F’, with equal frequency) would appear randomly in the location previously occupied by a neutral expression or a disgusted expression with equal frequency. Participants were instructed to identify the probe as quickly as possible by pushing the controller joystick left (for ‘E’) or right (for ‘F’). A 500 ms inter-trial interval took place before a new trial began. *Note. The BP4D-Spontaneous database is proprietary, therefore the actual stimuli used are not permissible to print in publications. The faces shown in this example comes from the Umeå University Database of Facial expressions (Samuelsson et al., 2012). Examples of the VR environment (as seen on a computer monitor) can be found in Supplementary Materials.

The dot-probe task was used to measure attentional bias (see Fig. 1 for task description).

Trials in which the probe appeared behind the disgusted expression were *congruent*. Trials in which the probe appeared behind the neutral expression were *incongruent*. A bias index was calculated by comparing a participant's average reaction time in incongruent trials versus congruent trials.

$$\text{Bias index}=\text{Mean}\left({\mathrm{RT}}_{\text{incongruent}}\right)-\text{Mean}\left({\mathrm{RT}}_{\text{congruent}}\right)$$

A positive bias index indicated that the participant reacted faster to probes when they appeared behind disgusted faces, while a negative bias index indicated a faster reaction to probes behind neutral faces.

#### 2.3.4 Attentional bias modification (person-identity-matching task)

**Fig. 2:**
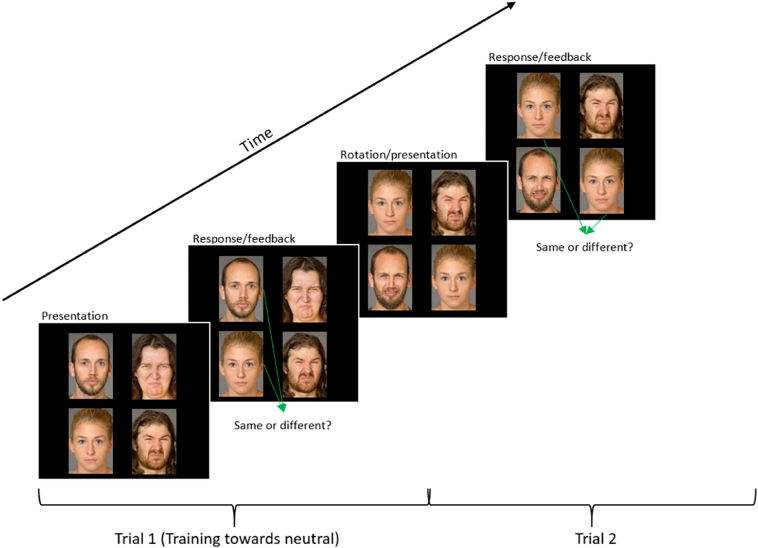
Example trial of a person-identity-matching task. Two pairs of faces were presented simultaneously, forming a 2 × 2 grid of 4 faces. Each pair consisted of two individuals, one displaying a neutral expression and the other expressing disgust. In the neutral variant of this PIM task, participants were instructed to ignore the faces with a disgusted expression and only pay attention to the neutral faces in order to identify whether the two neutral faces belonged to the same individual (identity match). The participants used the controller joystick to respond by pushing it left for ‘yes/same’ and right for ‘no/different’. If the participants responded correctly, the background of the VR environment would flash green, whereas if they responded incorrectly it would flash red. A new trial began with the top pair of faces rotating out of the grid, the bottom pair rotating to the top, and a new pair of faces appearing at the bottom.

Bias modification was carried out using a PIM task adapted from Notebaert et al. (2015); see Fig. 2 for task description).

In the neutral PIM, participants were instructed to identify whether the two neutral faces belonged to the same individual. Conversely, in the disgust PIM, participants were instructed to identify whether the two disgusted faces shown belonged to the same individual.

### 2.4 Procedure

Interested participants were registered and screened on the study website. Eligible participants were invited to book a VR session at Stockholm University, and provided written informed consent upon arrival. The participants were randomised to the four experimental groups (2D neutral, 2D disgust, 3D neutral, and 3D disgust). Before the VR session began, participants completed the pre-training assessment questionnaires (LSAS-SR, PHQ-9, GAD-7, DERS-16, and BBQ).

To minimise the impact of participant cancellation on data collection, randomisation was done on VR training sessions rather than individual participants. Sessions were pseudorandomised in blocks of 4, 8, or 12 to the four experimental groups using R. Since the order of group affiliation was pre-designated, whenever a participant failed to come to an appointment, the group affiliation for the cancelled session (and subsequent sessions) would transfer to the next participant. The VR data were linked to each participant by their participant ID, which the experimenters manually input into the programme at the start of the session. Since the experimenters also needed to select the correct task for each condition before training begins, they were not blind to the experimental conditions.

The VR session began with a quick visual acuity check inside the VR environment to ensure that all participants could see the images clearly. The participants then familiarised themselves with the dot-probe task by completing a tutorial (five consecutive correct responses to probes). Baseline attentional bias was measured using 100 trials of dot-probe task. All bias measurements were carried out using 2D stimuli, regardless of what stimuli were used in the PIM training.

After bias measurement, the participants underwent another tutorial to learn the PIM task. Upon successfully finishing the tutorial (five consecutive correct responses), they completed two blocks of ABM training (190 trials each) with a self-paced break between blocks. Depending on their group affiliation, the participants received ABM with: (i) 380 trials of disgust PIM with 2D stimuli; (ii) 380 trials of neutral PIM with 2D stimuli; (iii) 380 trials of disgust PIM with 3D stimuli; or (iv) 380 trials of neutral PIM with 3D stimuli. After the training phase, attentional bias was measured again using 100 trials of dot-probe. The participants finished the VR session by filling out the LSAS-SR again to assess their social anxiety post-training. Post-ABM questionnaires (LSAS-SR, PHQ-9, GAD-7, DERS-16, and BBQ) were sent to the participants for follow-up assessments at seven days and 90 days after the VR session.

### 2.5 Statistical analyses

All statistical analyses were performed in R (version 3.6.1; R Core Team, 2019).

## 3 Results

**Fig. 3:**
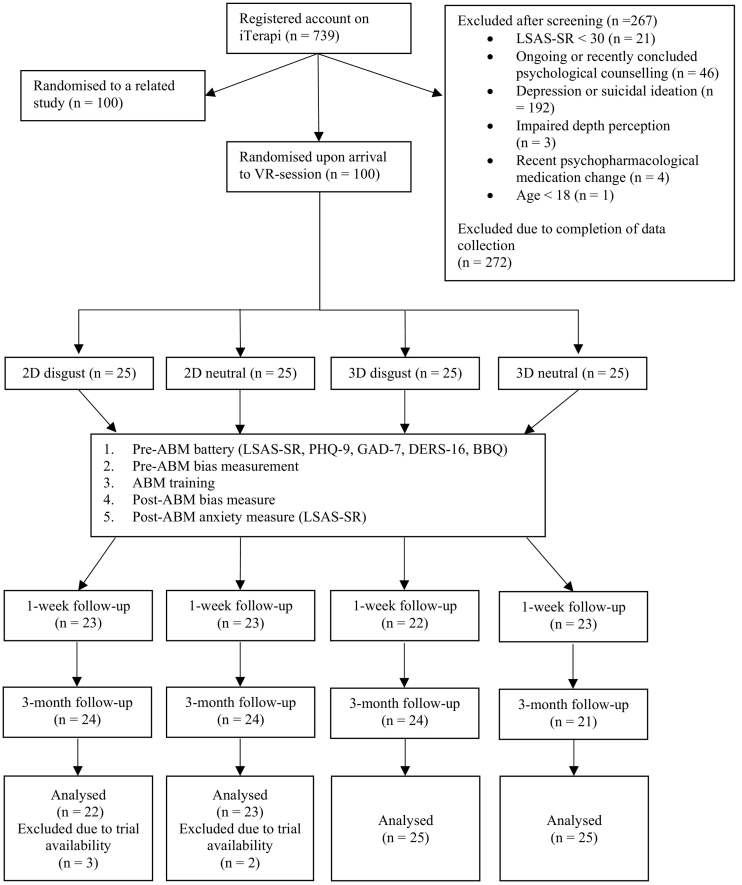
Overview of participant flow throughout the study.

All 100 participants completed pre-ABM and post-ABM assessment of anxiety and bias. For follow-up measures, nine participants failed to complete the 1-week follow-up and seven participants failed to complete the 3-month follow-up. For the bias measurement data, trials were discarded if they (i) were error trials; (ii) had a response time <200 ms or >2000 ms; or (iii) had a response time that was beyond 2 standard deviations from the individual's mean response for each trial type (congruent/incongruent). Five participants were excluded from analyses as they had more than 20% of their trials discarded for at least one of the trial types in either the pre- or the post-training bias measurement task (see Fig. 3; for details of the data cleaning procedure, please refer to analysis script). None of the groups differ on any demographic characteristics or measures at baseline except for bias index at pre-training (see Table 1).

**Table 1:** Participant demographics and characteristics at baseline.

		2D disgust (*N* = 22)	2D neutral (*N* = 23)	3D disgust (*N* = 25)	3D neutral (*N* = 25)	Between-groups comparison
Male	*N* (%)	8 (36%)	14 (61%)	11 (44%)	15 (60%)	χ^2^ = 4.07, *p* = .254
Tertiary education	*N* (%)	16 (73%)	14 (61%)	13 (52%)	14 (56%)	χ^2^ = 2.33, *p* = .508
Age	*M* (*SD*)	40.23 (13.53)	39.35 (12.40)	35.92 (10.88)	38.08 (13.08)	F_(3,91)_ = 0.53, *p* = .660
Bias index (ms)	*M* (*SD*)	9.47 (37.29)	−23.39 (48.28)	9.11 (37.72)	−2.21 (32.80)	F_(3,91)_ = 3.56, *p* = .017⁎
Liebowitz Social Anxiety Scale, Self-reported (LSAS-SR)	*M* (*SD*)	63.91 (20.61)	62.22 (18.15)	65.28 (27.37)	67.72 (23.52)	F_(3,91)_ = 0.25, *p* = .862
Patient Health Questionnaire (PHQ-9)	*M* (*SD*)	6.09 (3.60)	5.74 (3.43)	4.48 (3.50)	5.48 (4.10)	F_(3,91)_ = 0.85, *p* = .470
Generalised Anxiety Disorder 7-item scale (GAD-7)	*M* (*SD*)	4.64 (4.11)	4.96 (2.87)	4.68 (4.07)	5.40 (4.32)	F_(3,91)_ = 0.20, *p* = .898
Difficulties in Emotion Regulation Scale-16 (DERS-16)	*M* (*SD*)	37.50 (14.08)	31.74 (8.32)	36.00 (16.21)	39.76 (15.75)	F_(3,48.7)_ = 2.12, *p* = .110
Brunnsviken Brief Quality of Life Inventory (BBQ)	*M* (*SD*)	52.23 (16.76)	43.61 (16.12)	54.40 (26.62)	46.84 (18.65)	F_(3,50.3)_ = 1.50, *p* = .226

*Note*. ANOVAs were conducted for all other measures. Levene's test revealed that assumption of homogeneity of variances was violated for DERS-16 (*p* = .02) and BBQ (*p* = .02), thus these two ANOVAs were conducted without assumption of equal variances. For bias index, a Tukey's test revealed that the 2D neutral group had a significantly lower average bias index at baseline compared to the 2D disgust and 3D disgust groups.

⁎ *p* < .05. For between-groups comparison, Pearson's chi-squared tests were conducted for sex and education.

### 3.1 Association between bias and anxiety symptoms

**Fig. 4:**
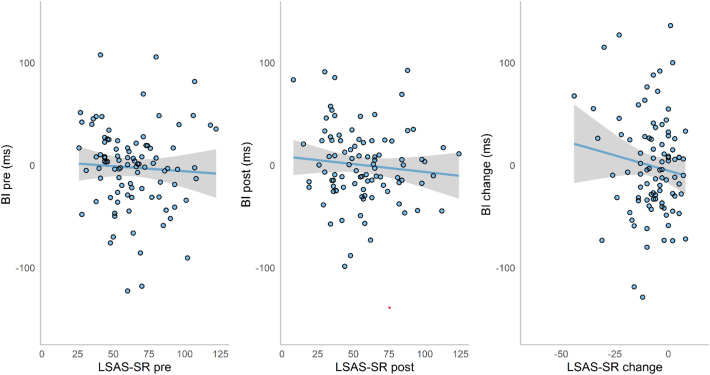
Scatterplots with trend lines of bias index (BI) and anxiety scores (LSAS-SR) at pre-ABM, post-ABM, and their changes over time. Shaded regions indicate 95% CI.

The relationship between attentional bias and anxiety symptoms was explored using simple correlations (Fig. 4). No significant correlations were found between bias index and LSAS-SR scores at pre-ABM (*r* = −0.02, *p* = .88) or post-ABM (*r* = 0.03, *p* = .78), or between bias change and anxiety score change (*r* = −0.08, *p* = .44).

### 3.2 Bias change

To evaluate whether the ABM training successfully induced a change in attentional bias, a 2 × 4 repeated measures ANOVA with the four groups as between-subjects factor and time (pre- vs. post-training) as within-subjects factor was performed. The results showed no significant difference in bias between pre- and post-training (F_(1,91)_ = 0.37, *p* = .544). No group differences were observed (F_(3,91)_ = 1.40, *p* = .248). No interaction between group and time was found (F_(3,91)_ = 1.34, *p* = .267). The result suggests that attentional bias did not change after ABM using the PIM task, regardless of training contingency or stimuli used.

**Fig. 5:**
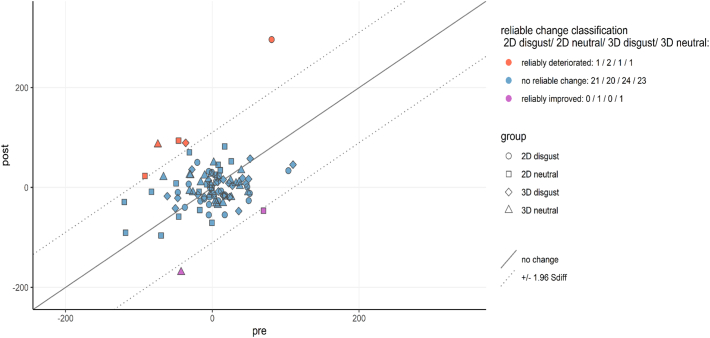
Reliable change plot for individual participant's bias index.

We further explored bias modification at the individual level by calculating the reliable change indices for each participant, using methods proposed by Jacobson and Truax (1991). First, the standard error of measurement (SE_M_) was calculated based on the sample baseline standard deviation and split-half reliability of the pre-training dot-probe task. The Spearman-Brown corrected average reliability estimate of 5000 random splits (Parsons, 2018) served as a measure of internal reliability for bias index. The resulting estimate was very low (*r* = 0.05). A Standardised Difference Score (S_diff_) was computed based on the standard error of measurement (S_diff_ = √(2*SE_M_^2^). If an individual's bias index was reduced by at least 1.96 times the S_diff_, they were classified as showing a reliably improved bias (i.e. reduced attentional bias towards threat). If an individual's bias index increased by at least 1.96 times the S_diff_, they were classified as showing a reliably deteriorated bias towards negative. If an individual's bias change fell within the range of 1.96 S_diff_, they were classified as unchanged. The results showed that five participants had a reliable deterioration in bias, 88 participants showed no reliable change, and only two participants achieved reliable improvement in their attentional bias after ABM training (Fig. 5).

### 3.3 Anxiety change

A mixed model approach was used to analyse how anxiety scores changed across the four groups over time. Two dummy-coded variables replaced the group variable to dissociate the effects of training condition (disgust = 0 vs. neutral = 1) and stimuli used (2D = 0 vs. 3D = 1). We used the nlme() package (Pinheiro and Bates, 2014) in R to compare different models on their fit to the data using the Akaike information criterion (AIC), with values ranging from 2986.3 (effect of time only) to 3353.8 (null model with intercept only). For details of model comparison, see Supplementary Materials.

**Fig. 6:**
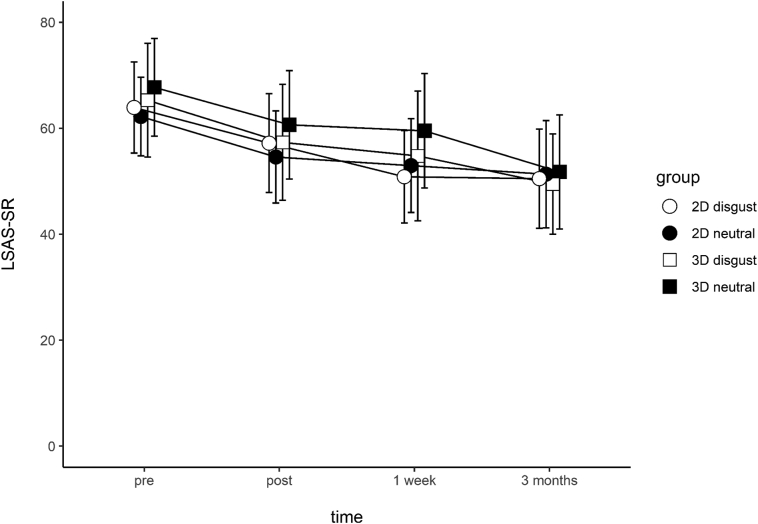
Liebowitz Social Anxiety Scale, Self-reported (LSAS-SR) score change across time. Error bars represent 95% CI.

A linear mixed-effects model analysis was carried out using the full model. Time, condition, 2D/3D stimuli, and all 2-way and 3-way interactions were modelled as fixed effects. Random intercepts and random slopes for each participant were modelled as random effects. For main effects, only time was significant – on average, participants showed a reduction of 4.6 points in their LSAS-SR score at each successive assessment (*t*_(265)_ = −3.45, *p* < .001, Nagelkerke's pseudo *r*^2^ = 0.65). No other main effects or interactions were significant (Table 2). Fig. 6 illustrates the LSAS-SR reduction over time, separated by groups.

**Table 2:** Fixed effects parameter estimates.

Effect	Estimate	SE	DF	*t*	*p*
Intercept	67.56	5.78	265	11.68	< 0.001⁎⁎⁎
Time	−4.57	1.10	265	−3.45	< 0.001⁎⁎⁎
Condition	−3.90	8.09	91	−0.48	0.63
2D/3D	1.66	7.93	91	0.21	0.83
Time x condition	1.14	1.85	265	0.61	0.54
Time x 2D/3D	−0.52	1.82	265	−0.29	0.77
Condition x 2D/3D	5.92	11.16	91	0.53	0.60
Time x condition x 2D/3D	−0.36	2.57	265	−0.14	0.89

⁎⁎⁎ *p* < .001.

Although a simpler model with only the main effect of time (*t*_(268)_ = −6.82, *p* < .001) had the lowest AIC value, directly comparing this model against the full model did not reveal a significantly better fit (likelihood-ratio = 1.75, *p* = .941).

### 3.4 Clinically significant change

Jacobson-Truax clinical change indices were computed for LSAS-SR scores. Here we applied the full clinical change index calculation (as opposed to just the reliable change calculation done for bias change). For each participant, reliable change was determined first (defined as change surpassing 1.96 S_diff_), followed by application of the A criterion to determine clinical change. The A criterion was based on the sample baseline distribution of LSAS-SR scores – participants whose post-training scores were lower than the baseline group mean score minus 1.96 times the baseline standard deviation were classified as ‘recovered’, indicating that their post-ABM scores fall outside the 95% confidence interval of the sample's distribution at baseline.

**Fig. 7:**
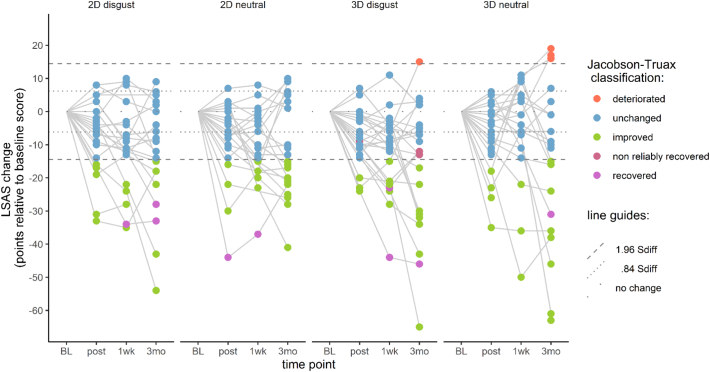
Jacobson-Truax (Criterion A) classification of clinically significant change across time.

For the calculation of the reliable change criterion, Cronbach's alpha was determined using the psych package (Revelle, 2018). The internal reliability of the LSAS-SR was found to be satisfactory (α = 0.95). Scores at post-training, 1-week, and 3-month follow-ups were all compared to baseline. At each time point, participants who showed reliable change (i.e. changed more than 1.96 S_diff_) and a score below the cut-off were classified as ‘recovered’. Participants who scored below the cut-off but did not show reliable change were classified as ‘non-reliably recovered’. Participants who showed reliable change but did not score below the cut-off points were classified as ‘improved’. Participants who did not show reliable change were classified as ‘unchanged’. Participants who showed reliable increase in LSAS-SR scores would have been classified as ‘deteriorated’. At the 3-month follow-up, four participants were classified as ‘recovered’, two ‘non-reliably recovered’, 34 ‘improved’, 44 ‘unchanged’, and four ‘deteriorated’ (Fig. 7).

### 3.5 Secondary outcome measures

All secondary outcomes were analysed using linear mixed models. None of the analyses revealed any significant effect, indicating that the ABM training did not have an impact on depression, generalised anxiety, emotional regulation or quality of life.

## 4 Discussion

The current study investigated the efficacy of a VR-based person-identity-matching task in reducing attentional bias and social anxiety in participants with LSAS-SR scores comparable to a clinical population. After a single session of training, we did not observe any changes in attentional bias. Contrary to our hypotheses, all groups showed reduction in anxiety symptoms post-training (*p* < .001, Nagelkerke's pseudo *r*^2^ = 0.65), regardless of group affiliation (neutral vs disgust PIM; 2D vs 3D stimuli). This reduction was maintained at the 1-week and 3-month follow-ups. At the 3-month follow-up, only 4% of participants met the ‘recovered’ criteria for clinically significant change, while anxiety scores were classified as ‘unchanged’ for 46% of the participants.

### 4.1 Failure to detect bias

The lack of evidence for attentional bias in socially anxious individuals has been reported in numerous recent studies (Boettcher et al., 2013; Heeren et al., 2015a; Ma et al., 2019; Miloff et al., 2015; for a recent meta-analysis of baseline bias in ABM RCTs, see Kruijt et al., 2019). In addition, we found no evidence of change in bias after ABM training using the PIM task (cf. Notebaert et al., 2015). One potential explanation for the failure to detect bias is the poor reliability of the dot-probe task (e.g. Chapman et al., 2017; Schmukle, 2005; Waechter and Stolz, 2015). The Spearman-Brown corrected split-half estimate for internal reliability for the bias index was extremely low (*r* = 0.05). A similarly low reliability estimate (*r* = −0.04) for bias index obtained using the dot-probe task from another sample of 100 participants in a related study conducted by our research group has been reported elsewhere (Ma et al., 2019). Therefore, developing reliable measures of attentional bias should be the top priority for ABM research (Huppert et al., 2018; Rodebaugh et al., 2016). Any attempt at achieving bias modification would be futile without a reliable way to measure bias.

### 4.2 Symptom reductions

There was an overall reduction in social anxiety scores post-ABM training across all participants, which was maintained at the 3-month follow-up. However, in terms of clinically significant change, only 4% (4 out of 95) of participants achieved the ‘recovered’ status. A number of studies have reported that both active ABM and mock ABM can induce similar levels of symptom reduction (e.g. Boettcher et al., 2013; Bunnell et al., 2013; Enock et al., 2014; McNally et al., 2013). These findings seem to suggest that even when ABM tasks failed to measure or change attentional bias, there might be components in the training procedures that produced therapeutic effect. The mechanism of this therapeutic effect is difficult to pinpoint. In our study, the lowered anxiety cannot be attributed to any specific training contingency or stimuli, because all groups exhibited similar levels of anxiety reduction. It has been proposed that nonspecific factors such as placebo effect resulted from participating in a study could also contribute to symptom reduction in ABM studies (Enock et al., 2014). Similarly, the exposure effect from viewing facial expressions throughout the VR session may have contributed to symptom reduction.

### 4.3 Limitations

The current study has a number of limitations that need to be taken into consideration when interpreting the results. Firstly, our finding of no bias change following PIM training is not in line with the original study by Notebaert et al. (2015). Using a novel task poses its own challenges when it comes to interpreting the results, and the comparison to the original study is further complicated by the change in facial expressions (i.e. neutral vs. disgusted instead of happy vs. angry), the lack of a stressor task, and the introduction of VR. An a priori power analysis performed during the planning stages of the study showed that to detect a medium effect size (f = 0.25) and an alpha of 0.5, a total sample size of 100 was sufficient to achieve 80% power. However, it has been suggested that effect sizes for ABM might be larger for stressor vulnerability than symptom reduction (Jones and Sharpe, 2017). Therefore, it is possible that the current study does not have a large enough sample size to detect ABM effects, especially in the absence of a stressor task. Secondly, all outcome measures except for attentional bias were self-reported. The participants in our study were not assessed by formal diagnostic criteria, thus cannot be considered a clinical population. However, the average LSAS-SR score at baseline was 65, which indicated probable SAD diagnosis (Rytwinski et al., 2009). Thirdly, the facial expressions used in the current study were not validated. Anecdotal reports from experimenters during data collection noted how some participants perceived the negative facial expressions as ‘angry’ instead of ‘disgusted’. Fourthly, since the current study lacks a wait list control group, it is difficult to discern whether symptom reduction was ABM-specific, or due to factors such as spontaneous recovery or placebo effect stemming from taking part in a clinical study.

## 5 Conclusion

To summarise, a single-session, VR-based PIM task did not result in ABM-specific reduction in attentional bias or anxiety symptom. Both training towards neutral and training towards disgust achieved similar levels of anxiety reduction, which was maintained at 3-month follow-up. The anxiety reduction could not be attributed to changes in attentional bias, as we failed to detect bias at baseline, nor could we change bias with ABM training. More accurate, reliable, and precise measures of attentional bias are needed before we can properly assess the efficacy of any ABM procedure.

## Open science and pre-registration

We strive to adhere to the principles of Open Science. Unfortunately, the current study was not pre-registered before the commencement of data collection. In our effort to best compensate for the lack of pre-registration, all data used in the current study, as well as the complete R script used for data cleaning and analyses will be made openly accessible.

## Declarations of competing interest

The authors declare no conflict of interest.

## Appendix A Supplementary data

Supplementary Material 2 Screenshots of VR environment. Supplementary Material 2

Supplementary Material 3 Self-reported measures. Supplementary Material 3
