# Supplementary material for: Seeking neutral: A VR-based person-identity-matching task for attentional bias modification – A randomised controlled experiment
Source: Internet Interv. 2020 Aug 14;21:100334. doi: 10.1016/j.invent.2020.100334 (PMC7452567; doi:10.1016/j.invent.2020.100334)
Supplement: The following are the supplementary data related to this article.Supplementary Material 1 — Mixed-effects model comparison. [file mmc1.docx]

*Mixed-Effects Model Comparison*

| **Model** | **Model component** | | | **AIC** |
| --- | --- | --- | --- | --- |
|  | **Main effect** | **Interaction** | **Random effect**  **(participant)** |  |
| 1 | intercept only (null model) | - | - | 3353.8 |
| 2 | null model | - | intercept | 3081.9 |
| 3 | null model | - | intercept and slope | 3022.3 |
| 4 | time | - | intercept and slope | **2986.3** |
| 5 | condition | - | intercept and slope | 3024.1 |
| 6 | time + condition | time * condition | intercept and slope | 2989.6 |
| 7 | time + condition + stimuli | all 2-way interactions | intercept and slope | 2994.5 |
| 8 | time + condition + stimuli | all 2-way interactions and 3-way interaction | intercept and slope | **2996.5** |

**Note.* Model 1 was fitted with generalised least squares (gls) with maximum likelihood (ML). Models 2-8 were fitted with linear mixed-effects (lme) with ML.
