# Supplementary material for: Seeking neutral: A VR-based person-identity-matching task for attentional bias modification – A randomised controlled experiment
Source: Internet Interv. 2020 Aug 14;21:100334. doi: 10.1016/j.invent.2020.100334 (PMC7452567; doi:10.1016/j.invent.2020.100334)
Supplement: Supplementary Material 2 — Screenshots of VR environment. [file mmc2.pdf]

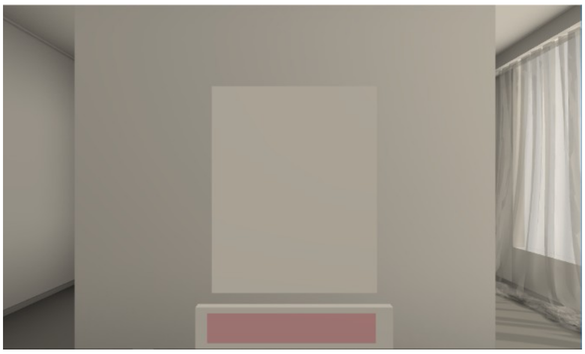

(a) VR environment

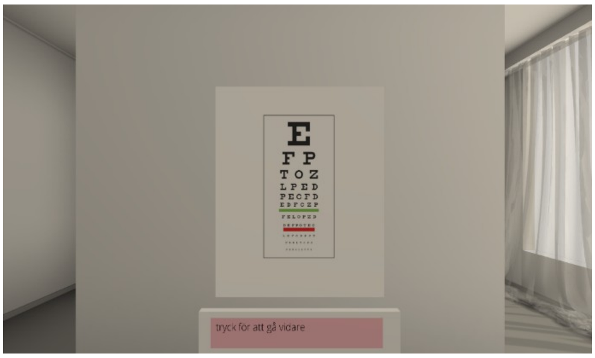

(b) Snellen chart for visual acuity check

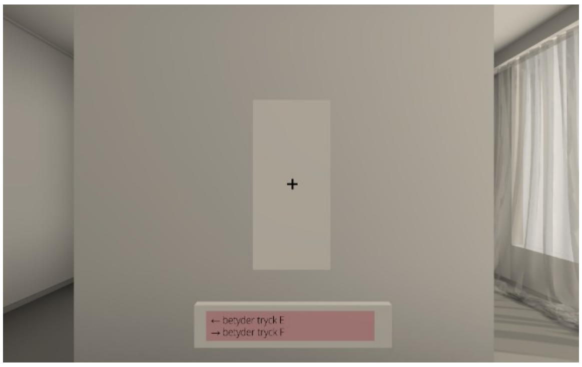

(c) Fixation cross

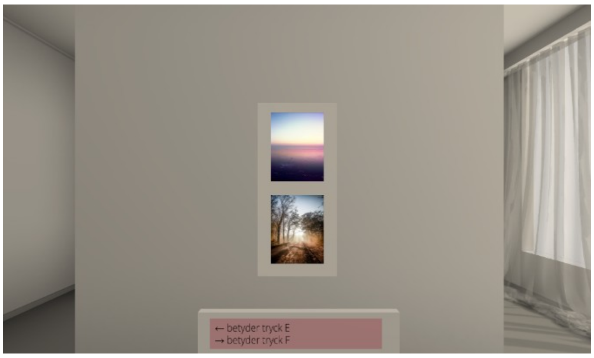

(d) Stimuli presentation

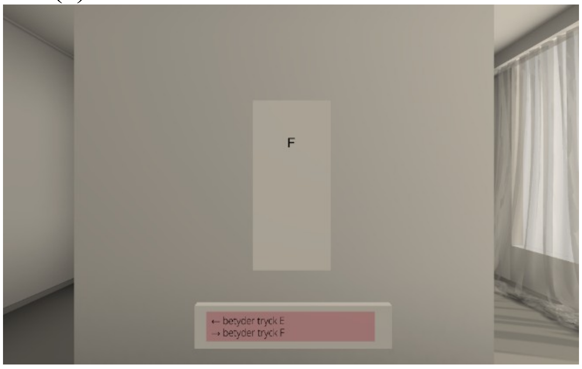

(e) Probe presentation

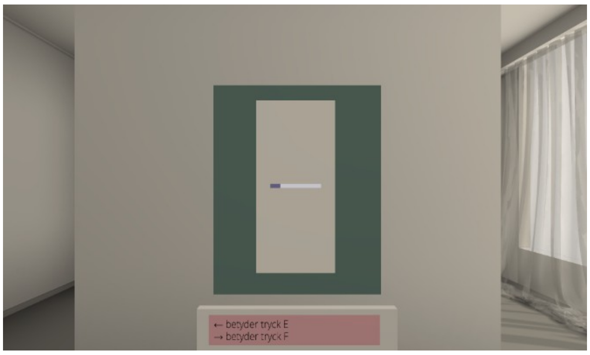

(f) Response feedback (tutorial only)
