# Supplementary material for: Seeking neutral: A VR-based person-identity-matching task for attentional bias modification – A randomised controlled experiment
Source: Internet Interv. 2020 Aug 14;21:100334. doi: 10.1016/j.invent.2020.100334 (PMC7452567; doi:10.1016/j.invent.2020.100334)
Supplement: Supplementary Material 3 — Self-reported measures. [file mmc3.docx]

*Summary of Self-Reported Measures Across Time, Separated by Groups*

|  | **Group** | **2D disgust**  ***M (SD)*** | **2D neutral**  ***M (SD)*** | **3D disgust**  ***M (SD)*** | **3D neutral**  ***M (SD)*** |
| --- | --- | --- | --- | --- | --- |
|  | ***N*** | **22** | **23** | **25** | **25** |
| ***Liebowitz* *Social* *Anxiety* *Scale*, *Self*-*reported*** | | | | | |
| Pre |  | 63.91 (20.61) | 62.22 (18.15) | 65.28 (27.37) | 67.72 (23.52) |
| Post |  | 57.18 (22.33) | 54.57 (21.29) | 57.32 (27.93) | 60.64 (26.08) |
| 1-week |  | 50.82 (19.92) | 52.95 (20.71) | 54.77 (29.28) | 59.52 (26.45) |
| 3-months |  | 50.48 (21.91) | 51.32 (24.20) | 49.46 (23.68) | 51.76 (25.18) |
| ***Patient* *Health* *Questionnaire*** | | | | | |
| Pre |  | 6.09 (3.60) | 5.74 (3.43) | 4.48 (3.50) | 5.48 (4.10) |
| 1-week |  | 5.85 (3.69) | 4.95 (3.34) | 4.68 (4.85) | 4.91 (3.57) |
| 3-months |  | 5.90 (5.04) | 5.45 (4.26) | 4.46 (4.86) | 5.76 (5.00) |
| ***Generalised* *Anxiety* *Disorder* 7-*item* *scale*** | | | | | |
| Pre |  | 4.64 (4.11) | 4.96 (2.87) | 4.68 (4.07) | 5.40 (4.32) |
| 1-week |  | 4.05 (3.62) | 4.71 (2.95) | 4.14 (3.99) | 4.95 (4.27) |
| 3-months |  | 3.57 (3.09) | 4.91 (3.74) | 3.54 (4.06) | 5.76 (5.22) |
| ***Difficulties* *in* *Emotion* *Regulation* *Scale*-*16*** | | | | | |
| Pre |  | 37.50 (14.08) | 31.74 (8.32) | 36.00 (16.21) | 39.76 (15.75) |
| 1-week |  | 34.75 (15.31) | 28.19 (9.10) | 34.14 (15.53) | 37.27 (14.50) |
| 3-months |  | 34.00 (14.71) | 27.14 (7.58) | 30.04 (12.17) | 33.57 (11.69) |
| ***Brunnsviken* *Brief* *Quality* *of* *Life* *Inventory*** | | | | | |
| Pre |  | 52.23 (16.76) | 43.61 (16.12) | 54.40 (26.62) | 46.84 (18.65) |
| 1-week |  | 51.20 (19.14) | 43.86 (18.45) | 55.55 (26.89) | 50.14 (16.91) |
| 3-months |  | 52.81 (20.83) | 45.82 (22.07) | 57.08 (25.03) | 48.86 (18.64) |
